# The Mortality from Phthisis: The Question of Personal Infection

**Published:** 1913-12-20

**Authors:** 


### THE MORTALITY FROM PHTHISIS.

### The Question of Personal Infection.

At the meeting of the Section of Epidemiology and State Medicine, Royal Society of Medicine, on Friday, November 28, Dr. W. H. Hamer, president, in the chair, Mr. E. C. Snow, D.Sc., read a very interesting paper entitled "The Mortality from Phthisis: a Statistical Investigation having Bearing upon the Question of Personal Communicability." He said the statistical methods of analysis developed in the past few years had so far only skimmed the immense storehouse of medical data extant. Nobody competent to express an opinion could doubt the possibility of personal infection in the case of phthisis, yet it could not be denied that susceptibility to, and possible immunity from, the tubercle bacillus varied according to hereditary disposition.

He had set himself to inquire how personal communicability of phthisis varied with age, and whether it varied with sex. These questions needed to be discussed from two points of view: (1) the power to infect, (2) susceptibility to infection. He had limited his investigation to the age period 15 to 45. It was necessary to have figures referring to males and females separately, and on the returns available he contended he had demonstrated that phthisis mortality was heaviest among males in districts in which there was a relative abundance of females, but that no association was apparent between the mortality of females from phthisis and the relative abundance of males. Under certain ideal conditions—which were never actually realised—this strongly suggested the infective character of phthisis, and pointed to a sexual difference in the manner of infection. So many possible causes were operating, all at the same time, in producing variations in the amount of phthisis, that only by rendering them constant in turn and considering the effects of a few only could the real causes of the variation be ascertained. Such factors were overcrowding, opportunity for infection, hereditary disposition, amount of institutional segregation, occupation, etc., and to find the effect of any one, populations should be selected for which the other factors were constant, and this could only be done by the statistical artifice of partial correlation. The author proceeded to evolve his thesis with great elaboration, and suggested that the results warranted the following conclusions: (1) The mortality from phthisis of males in any age-group is positively associated with excess of females in all age-groups. (2) There does not seem justification for concluding that an excess of males is, on the whole, accompanied by heavier phthisis mortality among the females. (3) An excess of young males is, on the whole, associated with heavier phthisis mortality among older males up to age 35, and similarly for females. An excess of older males, however, does not appear to be accompanied by heavier phthisis mortality among the younger males, and similarly also for the females.

There is definite evidence that the mortality of young males is associated with excess of females, and even of older females. There seems to be little evidence that the mortality of any age-group is associated with excess of males. That of young females is not associated with excess of females in other groups. Unless some other adequate explanation were forthcoming, he contended that personal communicability must be assigned as the cause, and this seemed to be operative principally in the earlier years of life. There was now a mass of statistical data on the subject, and opportunity was thus afforded of scrutinising more thoroughly than hitherto the relationship of phthisis to such factors as general environment and the degree of institutional segregation.

